# Supplementary material for: Experimental Trapped-ion Quantum Simulation of the Kibble-Zurek dynamics in momentum space
Source: Sci Rep. 2016 Sep 16;6:33381. doi: 10.1038/srep33381 (PMC5025896; doi:10.1038/srep33381)
Supplement: Supplementary Information [file srep33381-s1.pdf]

Supplementary material: Experimental Trapped-ion Quantum Simulation of the Kibble-Zurek dynamics in momentum space

Jin-Ming Cui,[1,2,*] Yun-Feng Huang,[1,2,*] Zhao Wang,[1,2] Dong-Yang Cao,[1,2] Jian Wang,[1,2] Wei-Min Lv,[1,2] Le Luo,[3,†] Adolfo del Campo,[4] Yong-Jian Han,[1,2,‡] Chuan-Feng Li,[1,2,§] and Guang-Can Guo[1,2]

[1] *Key Laboratory of Quantum Information, University of Science and Technology of China, CAS, Hefei, 230026, People's Republic of China*

[2] *Synergetic Innovation Center of Quantum Information and Quantum Physics, University of Science and Technology of China, Hefei, Anhui 230026, People's Republic of China*

[3] *Trapped Atoms and Ions Laboratory, Department of Physics, Indiana University-Purdue University Indianapolis, Indianapolis, IN 46202-3273*

[4] *Department of Physics, University of Massachusetts Boston, 100 Morrissey Boulevard, Boston, MA 02125*

(Dated: July 28, 2016)

The Kibble-Zurek mechanism is the paradigm to account for the nonadiabatic dynamics of a system across a continuous phase transition. Its study in the quantum regime is hindered by the requisite of ground state cooling. We report the experimental quantum simulation of critical dynamics in the transverse-field Ising model by a set of Landau-Zener crossings in pseudo-momentum space, that can be probed with high accuracy using a single trapped ion. We test the Kibble-Zurek mechanism in the quantum regime in the momentum space and find the measured scaling of excitations is in accordance with the theoretical prediction.

SINGLE TRAPPED-ION QUBIT

The ion qubit is manipulated as in [1], which is illustrated in Extended Data Figure 1A. Three lasers are used for Doppler cooling, state initialization and detection, respectively, which are generated by modulating 369 nm laser with different EOMs. After Doppler cooling for 3 ms, the ion nuclear spin of $S_{1/2}$ is initialized to the state $|0\rangle$ ($|F=0,\, m_F=0\rangle$) by a 100 μs pumping interval. Microwave for qubit operations drives the initialized state to the superposition state of $|0\rangle$ and $|1\rangle$ ($|F=1,\, m_F=0\rangle$), then the state is detected in 1 ms by exciting the $|1\rangle$ state with the detection laser and recording fluorescence photons with a counter. A histogram of detected photon number per run is depicted in Extended Data Figure 1B. When the ion is prepared in $|0\rangle$, the probability of detecting less than two photons in 99.35%. When the ion is prepared in $|1\rangle$ state, two or more photons are detected in 98.35%. Thus, when setting the threshold between 1 and 2 detected photons, the state detection fidelity is 98.85%, with a preparation and detection error of 1.15%. By varying the microwave driving time, Rabi oscillation between $|0\rangle$ and $|1\rangle$ can be measured, see Extended Data Figure 1C. The coherence time T_1 of the qubit is measured to be 77 ms by a damped sine curve fit of the oscillation data.

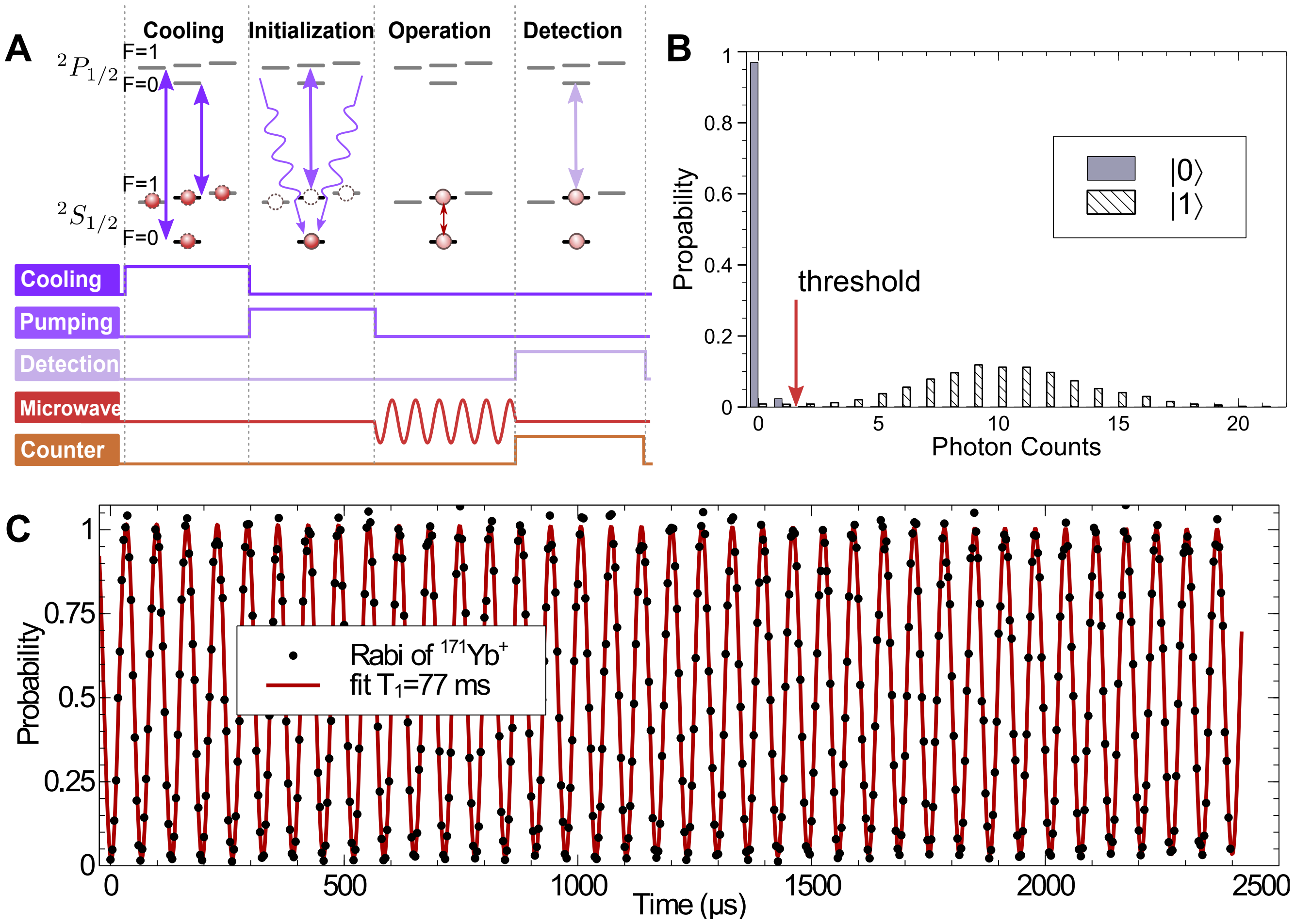

Extended Data Figure 1. **Qubit performance of the trapped ion.** (**A**) Control sequence of experiment. (**B**) Results of qubit state preparation and measurement, $|0\rangle$ and $|1\rangle$ were measured respectively, with state detection error of 1.15%. (**C**) Rabi oscillation during 2.4 ms, a coherent time of T_1 of 77 ms is determined by fitting the damping of the oscillation.

* These two authors contributed equally
† leluo@iupui.edu
‡ smhan@ustc.edu.cn

§ cfli@ustc.edu.cn

[1] S. Olmschenk, K. C. Younge, D. L. Moehring, D. N. Matsukevich, P. Maunz, and C. Monroe, Phys. Rev. A **76**, 052314 (2007).
